# “首届国际呼吸内镜诊治大会暨肺癌诊疗新技术新进展学习班”会议纪要

**DOI:** 10.3779/j.issn.1009-3419.2011.07.13

**Published:** 2011-07-20

**Authors:** 润波 钟

**Affiliations:** 200030 上海，上海交通大学附属胸科医院肺科 Department of Pulmonary, Shanghai Chest Hospital Affiliated to Shanghai Jiao Tong University, Shanghai 200030, China

由上海交通大学附属胸科医院、上海交通大学医学院主办，上海市医学会肺癌学组和卫生部呼吸内镜培训基地承办，2011年6月24日-6月26日在上海胸科医院及光大会展中心举办了为期两天半的“首届国际呼吸内镜诊疗大会暨肺癌诊疗新技术新进展学习班”。会议内容包括呼吸内镜介入技术在肺癌早期诊断和治疗、纵隔淋巴结诊断和分期中的运用、肺癌多学科治疗策略、肺癌小标本的采集与检测及分子靶向治疗在肺癌中的应用等。

本次会议邀请了廖美琳教授和吴一龙教授作为大会荣誉主席，大会主席由周清华教授、石远凯教授、韩宝惠教授、David Jablons教授和冯运院长担任。

本次盛会我们邀请到了来自美国的David Jablons、David Garfield、Michael Shurin、Galina Shurin、徐志东、徐莉教授，加拿大的Kazuhiro Yasufuku教授，德国的Felix JF Herth、Christian Manegold教授，日本的Noriaki Kurimoto教授，中国香港的林冰教授，还邀请了廖美琳、白春学、陈海泉、傅小龙、韩宝惠、李强、李时悦、石远凯、宋恕平、王长利、王贵齐、王洁、于金明、张力、张杰、支修益、周彩存、周清华等，专家进行了气管镜和肺癌相关领域的授课。

主会场首先由Kazuhiro Yasufuku、Felix JF Herth、Noriaki Kurimoto进行了凸面支气管镜的临床应用、径向支气管内超声及超声内镜在周围型病变中的应用的讲座，Christian Manegold、David Jablons、Michael Shurin等分别进行了非小细胞肺癌分子标记物意义和二线化疗及肺癌免疫治疗的专题讲座。

6月25日在气管镜分会场，Kazuhiro Yasufuku、Felix JF Herth在医院进行了超声内镜的操作演示，并在会场进行同时转播，香港的林冰教授进行现场解说。Yasufuku教授是第一个将EBUS应用于纵隔淋巴结活检及肺癌分期的学者，他与Herth教授的EBUS-TBNA操作给与会者留下了极深的印象，不仅操作极为规范，而且非常简练，穿刺检查后当场进行涂片镜检，如果阳性即可停止下一步继续操作，阴性则继续检查直至有阳性结果。这样的操作不仅在最大程度上缩短了患者行气管镜时所需耐受时间，也大大提高了诊断的阳性率。张杰和王贵齐教授紧接着进行了气道恶性肿瘤和EBUS-GS的现场操作，使与会者对国内外高水平的内镜操作有了最直接的感官认识。6月26日李强、张杰、李时悦、林冰、孙加源、王贵齐等6位专家先后针对气道支架植入、气道肿瘤热消融技术、气道肿瘤的腔内药物治疗、经支气管镜肺减容术、超声支气管镜及食道超声技术进展分别做了精彩的学术报告，与会代表通过答疑互动等多种形式就各自的学术见解及专业问题进行了充分的沟通和交流。

随着肺癌的发病率不断上升，肺癌的个体化治疗和靶向治疗已经成为当下的热点，如何使肺癌患者获得更好的生存时间以及如何更为早期的诊断和合理治疗肺癌是国内外学者研究的重点。2011年美国肿瘤年会（American Society of Clinical Oncology, ASCO）关于肺癌诊治的热点在于肺癌小病灶标本的获取和对于肺癌一线化疗耐药患者仍有必要尽可能获得组织标本以进行组织的分子分型。在通常肺癌的诊断中，往往因为创伤性较大或患者不愿意接受，肺癌组织标本的获取相对较为困难，而目前的观点认为应尽可能获取肺癌组织标本进行分子分型，进行基因突变的检测，以确定患者存在哪一种突变以及是否是靶向治疗的获益人群，以便在后续治疗中采取最适合患者的个体化治疗，达到“量体裁衣”的目的。在本次会议中，邀请了韩宝惠、白春学、周清华、王长利、陈海泉、张力、周彩存、支修益、王洁、于金明、石远凯、傅小龙、徐志东、徐莉、David Garfield、Galina Shurin等相应各领域的权威专家对于标本的分型及放疗、化疗、免疫治疗、靶向治疗进展，肺癌信号通路传导，个体化治疗及治疗耐药后的对策进行了细致的专题讲座，对提高参会人员对于这方面的认识有很大帮助。

本次大会中的专题学术讲座内容丰富新颖，代表着国内外的先进水平和未来研究方向，受到了与会代表的一致认可与赞扬，学术氛围热烈，这是一次相当成功的呼吸内镜和肺癌诊治大会。大会同时与国外相关领域专家进行了进一步深入交流，为今后在支气管内镜和肺癌领域的合作奠定了基础。

